# BCG and BCGΔBCG1419c protect type 2 diabetic mice against tuberculosis via different participation of T and B lymphocytes, dendritic cells and pro-inflammatory cytokines

**DOI:** 10.1038/s41541-020-0169-6

**Published:** 2020-03-12

**Authors:** Cristian Alfredo Segura-Cerda, Brenda Marquina-Castillo, Vasti Lozano-Ordaz, Dulce Mata-Espinosa, Jorge Alberto Barrios-Payán, Manuel O. López-Torres, Michel de Jesús Aceves-Sánchez, Helle Bielefeldt-Ohmann, Rogelio Hernández-Pando, Mario Alberto Flores-Valdez

**Affiliations:** 1 Doctorado en Farmacología, Universidad de Guadalajara, Sierra Mojada 950, Col. Independencia Oriente, 44340 Guadalajara, Jalisco Mexico; 2 Biotecnología Médica y Farmacéutica, Centro de Investigación y Asistencia en Tecnología y Diseño del Estado de Jalisco, A. C., Av. Normalistas 800, Col. Colinas de la Normal, 44270 Guadalajara, Jalisco México; 3 Laboratorio de Patología Experimental. Instituto Nacional de Ciencias Médicas y Nutrición Salvador Zubirán, Vasco de Quiroga 15, Belisario Domínguez sección 16, Tlalpan, Ciudad de México México; 4 School of Chemistry & Molecular Biosciences, University of Queensland St. Lucia Campus, St Lucia, QLD 4072 Australia

**Keywords:** Vaccines, Live attenuated vaccines

## Abstract

Comorbidity between Tuberculosis (TB) and type 2 diabetes (T2D) is one of the greatest contributors to the spread of *Mycobacterium tuberculosis (M. tuberculosis)* in low- and middle-income countries. T2D compromises key steps of immune responses against *M. tuberculosis* and it might affect the protection afforded by vaccine candidates against TB. We compared the protection and immune response afforded by the BCGΔBCG1419c vaccine candidate versus that of wild-type BCG in mice with T2D. Vaccination with both BCGΔBCG1419c, BCG or infection with *M. tuberculosis* reduced weight loss, hyperglycemia, and insulin resistance during T2D progression, suggesting that metabolic changes affecting these parameters were affected by mycobacteria. For control of acute TB, and compared with non-vaccinated controls, BCG showed a dominant T CD4^+^ response whereas BCGΔBCG1419c showed a dominant T CD8^+^/B lymphocyte response. Moreover, BCG maintained an increased response in lung cells via IFN-γ, TNF-α, and IL-4, while BCGΔBCG1419c increased IFN-γ but reduced IL-4 production. As for chronic TB, and compared with non-vaccinated controls, both BCG strains had a predominant presence of T CD4^+^ lymphocytes. In counterpart, BCGΔBCG1419c led to increased presence of dendritic cells and an increased production of IL-1 β. Overall, while BCG effectively reduced pneumonia in acute infection, it failed to reduce it in chronic infection, whereas we hypothesize that increased production of IL-1 β induced by BCGΔBCG1419c contributed to reduced pneumonia and alveolitis in chronic TB. Our results show that BCG and BCGΔBCG1419c protect T2D mice against TB via different participation of T and B lymphocytes, dendritic cells, and pro-inflammatory cytokines.

## Introduction

Tuberculosis (TB) remains as the first cause of death by a single infectious agent worldwide, as in 2018, TB caused 1.6 millions deaths and 10 million new cases^1^. Recent meta-analysis reported that T2D patients have a twofold to fivefold higher risk to develop TB compared with patients without T2D^2,3^. T2D coexist in 16% of the newly diagnosed TB cases, and it is estimated that 4.1% of T2D patients will eventually develop TB^4^. Current T2D prevalence rounds 425 million cases, and may increase to 629 million people in 2045^5^.

T2D generates a series of changes that compromises the host response against *M. tuberculosis*, as recently reviewed by some authors^6,7^, including altered capacity to present antigens by macrophages^8^, activation of monocytes^9^, and chemoattraction of immune cells to lung during infection^10^. Furthermore, T2D favors more severe manifestations of TB as compared with those occurring in patients without T2D^11^, including extended lung damage especially at chronic stages of the comorbidity^12,13^. Despite this, the evaluation of new treatments or preventive measures against TB in the context of T2D constitutes a poorly explored area^14^, with no report available as of today about the efficacy of protection of any vaccine candidate against TB in the context of T2D.

The BCGΔBCG1419c mutant strain, a BCG Pasteur derivative, has been shown to reduce lung pathology at the chronic stage in two murine models of TB^12,15^, and also reduced reactivation after immusuppression induced by corticosterone administration in a mouse model of latent TB infection (LTBI)^12^. Furthermore, BCGΔBCG1419c reduced lung damage at 6 months post reactivation from latent lymphatic TB^16^.

We hypothesized that given the capacity of BCGΔBCG1419c to reduce lung pathology in nondiabetic mice, it could be more effective than BCG to afford protection against *M. tuberculosis* in T2D conditions, and evaluated its efficacy at acute (2 months), and chronic (4 months) infection with *Mycobacterium tuberculosis* H37Rv in mice with T2D. We went further on to characterize the contribution of immune cells and key cytokines involved in TB protection and pathology.

## Results

### Vaccination with BCGΔBCG1419c reduces insulin resistance and body weight loss post infection as compared with BCG in T2D mice

**Fig. 1:**
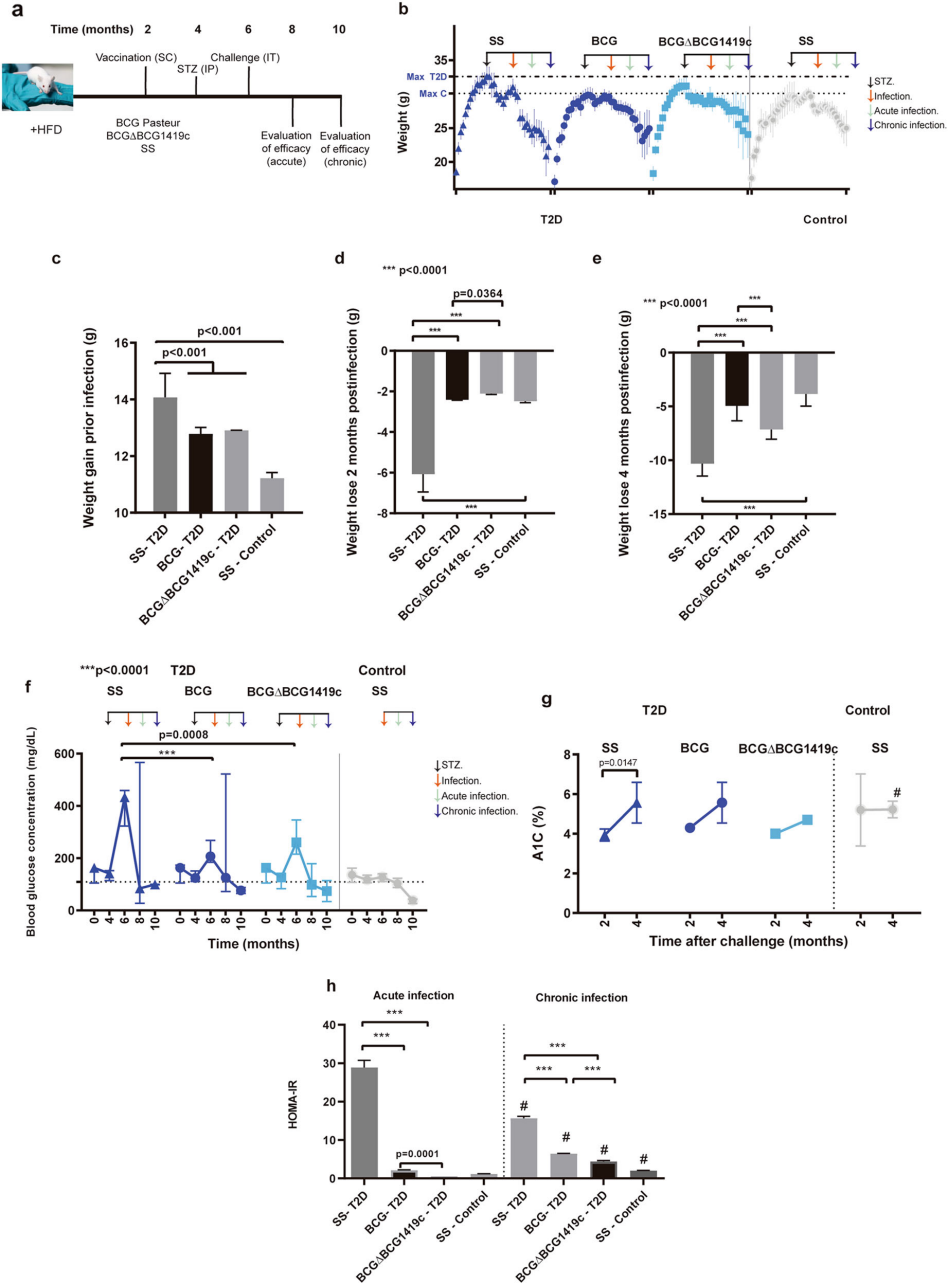
Progression of mice to T2D, vaccination and challenge. **a** T2D mice were fed with a HFD during the whole experiment, and control mice were feed with a balanced diet to control and determine metabolic parameters to confirm T2D. After 2 months of the beginning of HFD, mice progressing to T2D were injected intraperitoneally with 100 mg/kg of STZ to establish the T2D model. Prior to challenge with *M. tuberculosis* H37Rv, blood samples were taken for metabolic evaluation. Two and four months after infection, efficacy of protection of both BCG strains was assessed. **b** Mice were weighted weekly to monitor their weight gain before infection, or their weight loss after infection. Data are presented as mean and standard derivation of *n* = 25 mice per group. **c** Weight gain in mice of the groups prior to infection. Data are presented as median and range of all mice per time. **d**, **e** Weight loss in mice of the groups 2 and 4 months after infection. **f** Glucose concentration of mice was measured at 0, 2, 4, 6, and 8 months to confirm hyperglycemia and the later effect of vaccination and/or infection. Data are presented as median and range of 5 mice per time evaluated. **g** Hemoglobin A1C (A1C) in mice at 2 and 4 months after infection. Data are presented as median and range of 4 mice per time evaluated. **h** As an indicator of insulin resistance, HOMA-IR index was calculated for mice. Median and standard derivation of three calculations of HOMA-IR index is shown. Group comparisons where *p* < 0.05 were considered different. Data correspond to a representative replicate of two independent experiments. ***p < 0.0001 in the comparison between groups determined by ANOVA with Bonferroni correction for multiple comparisons; ^#^*p* < 0.05 in the comparison of the same group at 2 and 4 months after infection determined by a two-tailed Student’s *t* test.

T2D is characterized by the progressive development of insulin resistance and a reduced β-cell function^17^. To evaluate whether we established a T2D model in high-fat diet (HFD) plus streptozotocin (STZ)-treated mice (Fig. 1a), we monitored body weight weekly, blood glucose concentration, insulin, and determined the homeostasis model assessment of insulin-resistance (HOMA-IR) index at 2 and 4 months post challenge.

Prior to infection, T2D mice gained more weight than control mice (Fig. 1b, c), similar to what is observed in obese patients, who have a higher risk to progress to T2D^17^. In T2D mice, BCG- and BCGΔBCG1419c-vaccinated mice gained less weight than mock-vaccinated mice (Fig. 1c), with no difference between either BCG strain tested. Food intake between vaccinated- and mock-vaccinated mice groups was similar (Supplementary Table 1). Vaccination reduced total body weight loss post infection as compared with non-vaccinated mice (Fig. 1d). Of note, at acute infection, mice vaccinated with BCG lost more weight than mice vaccinated with BCGΔBCG1419c (*p* = 0.0364, Fig. 1d), whereas at chronic infection, mice vaccinated with BCGΔBCG1419c lost more weight than BCG-vaccinated mice (*p* < 0.0001, Fig. 1e). These results suggest that vaccination with BCG strains differentially modulate weight loss or gain in acute and chronic TB in infected T2D mice.

A key feature of T2D is hyperglycemia^17^. Before administration of STZ, mice progressing to T2D were overweight, but their blood glucose levels were equivalent to those in control mice (Fig. 1f). After administration of STZ to the HFD mice, glucose concentration was considerably higher in control mice (*p* < 0.0001) than in vaccinated mice (Fig. 1f). Even though both BCG- and BCGΔBCG1419c-vaccinated mice had a reduced blood glucose concentration, there were not significant differences in this parameter between these groups (*p* = 0.6261). After infection with *M. tuberculosis*, mice vaccinated with both BCG and BCGΔBCG1419c had lower blood glucose concentrations than before infection, without significant differences between vaccinated groups (Fig. 1f).

Glycated hemoglobin (A1C) levels in blood allows for long-term follow-up of diabetic condition in T2D^18^. A1C levels did not differ between vaccinated and non-vaccinated mice at 2 and 4 months; Conversely, mock-vaccinated T2D mice were increased A1C during acute and chronic infection, with vaccination with either BCG tended to reduce it (Fig. 1g).

To determine whether insulin resistance was maintained or not after *M. tuberculosis* challenge, we estimated the HOMA-IR index in mice at 2 and 4 months after infection. HOMA-IR is a an indirect measure of the insulin resistance and function of β-cells^19^. In the absence of vaccination, T2D resulted in higher HOMA-IR index than control mice at both time points, while vaccination reduced the index as compared with mock-vaccinated T2D mice, being lower in BCGΔBCG1419c-vaccinated mice. Of note, during TB, BCGΔBCG1419c reduced more the HOMA index than BCG at both acute (*p* = 0.0001) and chronic TB (*p* < 0.0001) (Fig. 1h).

In humans, T2D often derives in dysregulation of lipids metabolism, with high level of triglycerides in sera without changes in total cholesterol^20^. In our model, T2D mice showed no difference in triglycerides or total cholesterol in blood as compared with control mice, with no influence of vaccination on these parameters either (Supplementary Fig. 1). Then, our murine T2D model recapitulates weight gain, hyperglycemia, elevated levels of A1C, and insulin resistance but not the dysregulation of lipids observed in human patients with this disease.

### Vaccination with BCGΔBCG1419c reduces immunopathology in T2D mice as compared with BCG during chronic TB

**Fig. 2:**
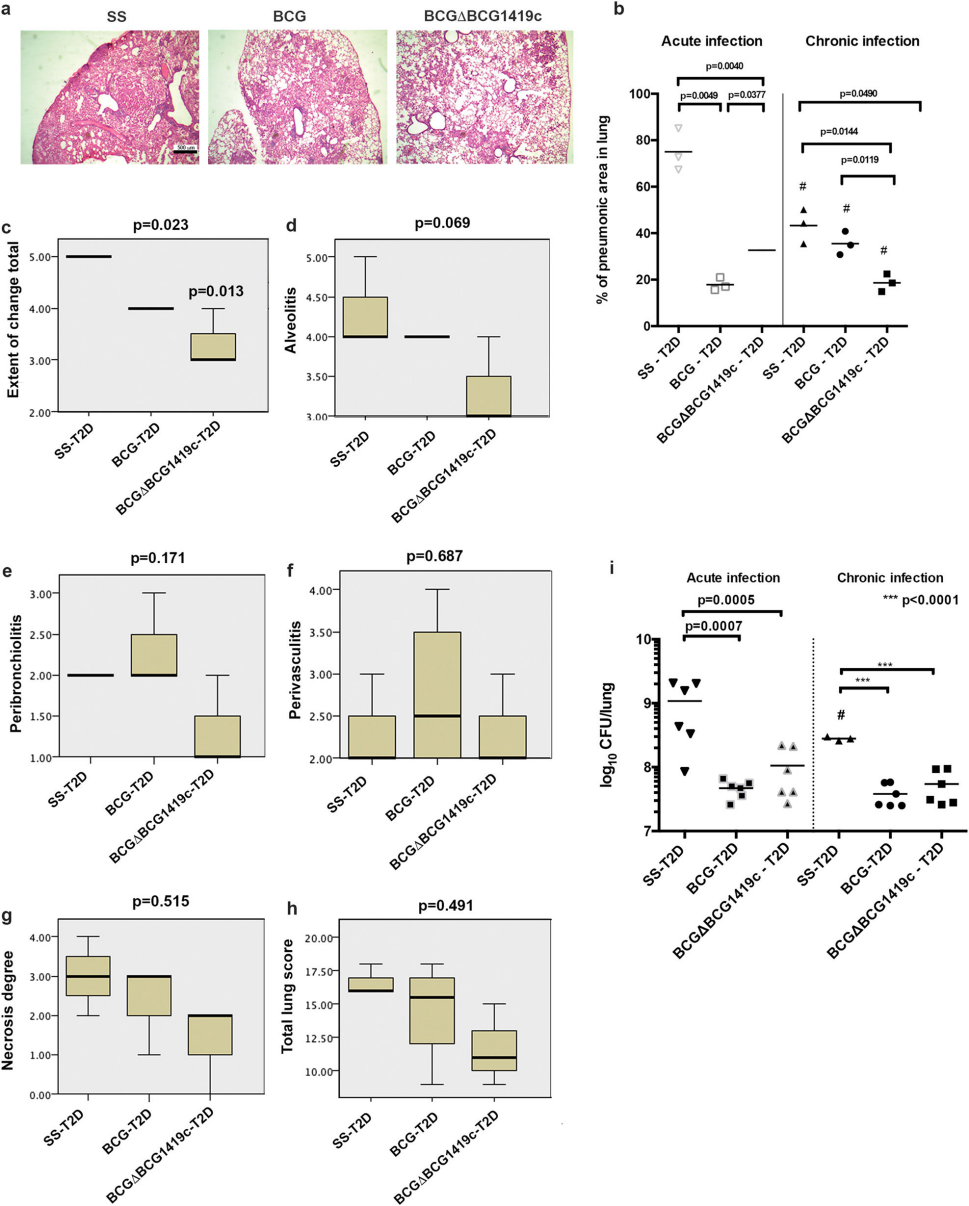
Vaccination with BCG or BCGΔBCG1419c reduces lung damage in T2D mice. Two months after the induction of T2D, mice were challenged with *M. tuberculosis* H37Rv and then evaluated at 2 and 4 months after infection. **a** Representative images of lung sections stained with hematoxylin and eosin, 2.5× at 4 months post infection. **b** BCG reduces lung pathology (percentage of pneumonic area in lung) 2 months after infection (acute TB) and also at 4 months after infection to a lesser degree, while BCGΔBCG1419c reduces the percentage of pneumonic area in lung at 4 months after infection (chronic TB) better than BCG. Pathological evaluation of (**c**) lung involvement (extent of change), (**d**) alveolitis, (**e**) peribronchiolitis, (**f**) perivasculitis, (**g)** necrosis, (**h**) total lung score, and (**i**) bacillary load in lung was evaluated at 2 and 4 months after infection. Graphs **c**–**i** show median value and rank. Group comparisons were *p* < 0.05 were considered different. Data correspond to a representative replicate of two independent in vivo experiments. ***p < 0.0001 in the comparison between groups determined by ANOVA with Bonferroni correction for multiple comparisons, and ^#^*p* < 0.05 in the comparison of the same group at 2 and 4 months after infection determined by a two-tailed Student‘s *t* test. For histological evaluation, data were compared using an H Kruskal–Wallis test with *p* values adjusted by Bonferroni correction.

Patients with TB-T2D often present extended lung damage especially at chronic stage of this comorbidity^11,17,21^. We found that T2D mock-vaccinated mice had a higher percentage of pneumonic area as compared with vaccinated mice, which reduced inflammation by twofold to fourfold (Fig. 2a, b). During acute TB, BCGΔBCG1419c-vaccinated T2D mice had a higher percentage of pneumonic area than BCG-vaccinated T2D mice (32.66 ± 6.22% vs. 17.86 ± 2.78%; *p* = 0.0377). When mice reached chronic TB, all groups of T2D showed a reduction of pneumonic area compared with the acute TB stage (indicated as # in Fig. 2a). Notably, BCGΔBCG1419c-T2D-vaccinated mice had a significant twofold lower percentage of pneumonic area than BCG- T2D-vaccinated mice (18.64 ± 3.18% vs. 35.50 ± 5.062%, respectively, *p* < 0.0119). Furthermore, only mice vaccinated with BCGΔBCG1419c had significantly reduced lung involvement (Fig. 2c, *p* = 0.013) with alveolitis approaching statistical significance (Fig. 2d, *p* = 0.069) but with no other difference detected for peribronchiolitis, perivasculitis, necrosis, or total lung score (Fig. 2e–h).

Regarding *M. tuberculosis* replication, at both acute and chronic infection, vaccination with either BCG or BCGΔBCG1419c resulted in a reduction in bacillary burden in lungs as compared with non-vaccinated mice, with no significant difference between BCG strains tested here (Fig. 2i), then showing that both BCG strains were equally effective in controlling *M. tuberculosis* H37Rv replication in lungs.

### Vaccination with BCG or BCGΔBCG1419c differently modifies the populations of immune cells in lungs of T2D mice with acute or chronic TB

**Fig. 3:**
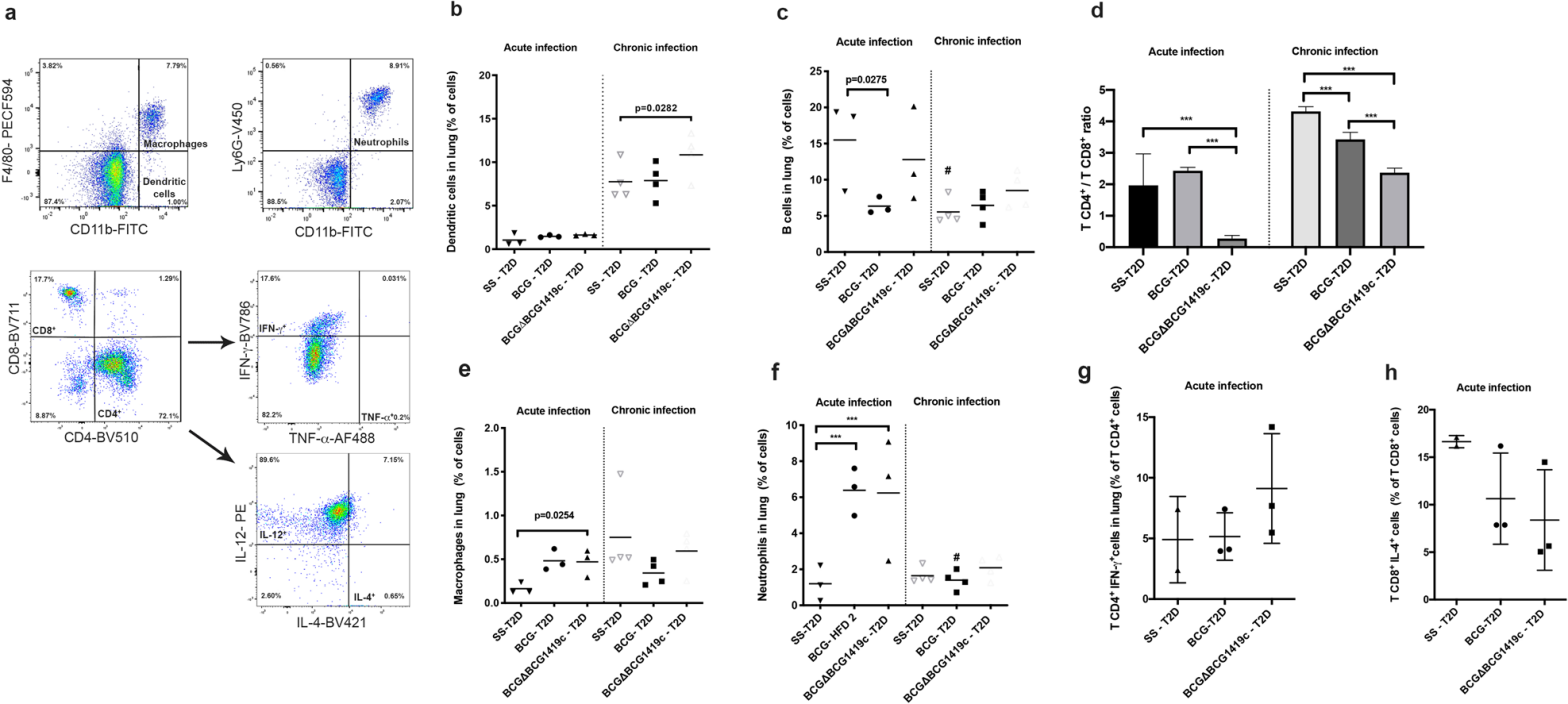
Lungs of T2D mice vaccinated with BCG or BCGΔBCG1419c strains show different proportion of T and B lymphocytes, and dendritic cells during active or chronic TB. Two and four months after challenge, lungs of mice were obtained and cell surface markers were used to identify populations of macrophages, dendritic cells, neutrophils, CD3^+^ CD4^+^, and CD3^+^ CD8^+^ T cells that produced cytokines by flow cytometry. **a** A representative gating strategy used to identify cells in lungs of vaccinated and non-vaccinated mice, T2D or control, at 2 or 4 months after challenge. **b** Percentages of dendritic cells found in non-vaccinated or BCG strains-vaccinated, T2D mice at 2 and 4 months after infection with *M. tuberculosis*. Dots represent individual values and the central line the mean. **c** Percentages of B cells found in non-vaccinated or BCG strains-vaccinated, T2D mice at 2 and 4 months after infection with *M. tuberculosis*. Dots represent individual values and the central line the mean. **d** T CD4^+^/T CD8^+^ cells ratio in non-vaccinated or BCG strains-vaccinated, T2D mice at 2 and 4 months after infection with *M. tuberculosis*. Bars represent mean values and standard derivation from eight samples per group. **e** Percentages of macrophages in lung of non-vaccinated or BCG-strains vaccinated, T2D mice at 2 and 4 months after infection with *M. tuberculosis*. Dots represent individual values and the central line the mean. **f** Percentages of neutrophils in lung of non-vaccinated or BCG-strains vaccinated, T2D mice at 2 and 4 months after infection with *M. tuberculosis*. Dots represent individual values and the central line the mean. **g** Percentages of CD4^+^ IFN-γ^+^ T cells in lung of non-vaccinated or BCG-strains vaccinated, T2D mice at 2 months after infection with *M. tuberculosis*. Dots represent individual values and the central line the mean. **h** Percentages of CD8^+^ IL-4^+^ T cells in lung of non-vaccinated or BCG-strains vaccinated, T2D mice at 2 months after infection with *M. tuberculosis*. Dots represent individual values and the central line the mean. Group comparisons were *p* < 0.05 were considered different. Data correspond to a representative replicate of two independent in vivo experiments. ***p < 0.0001 in the comparison between groups determined by ANOVA with Bonferroni correction for multiple comparisons; ^#^*p* < 0.05 in the comparison of the same group at 2 and 4 months after infection determined by a two-tailed Student’s t test.

Next, we evaluated whether T2D results in modifications of the recruitment of immune cells during acute and/or chronic *M. tuberculosis* infection and how vaccination with BCG strains would modify these changes (Fig. 3a). During acute infection, lungs from infected, mock-vaccinated T2D mice, had a 65.05 ± 12.45% of immune cells and vaccination with either BCG or BCGΔBCG1419c resulted in a reduction in the proportion of immune cells in lung (41.11 ± 1.44% and 43.43 ± 8.42%; *p* = 0.0088 and *p* = 0.0282 for the BCG or BCGΔBCG1419c vs. non-vaccinated controls, respectively). In T2D mice, we observed that vaccination with BCG strains had a different effect on the average composition of immune cells within lungs as compared with non-vaccinated mice, as follows: while BCG led to a 2.4-fold decrease of B lymphocytes (Fig. 3b, *p* = 0.0275), BCGΔBCG1419c avoided this reduction during acute infection (*p* = 1.000). On the other hand, BCG resulted in a T CD4^+^-biased response (CD4^+^/CD8^+^ ratio: 2.431 + 0.108) while BCGΔBCG1419c resulted in a T CD8^+^-biased response (CD4^+^/CD8^+^ ratio: 0.268 + 0.101, *p* < 0.0001, Fig. 3c). Both BCG strains induced increased migration of macrophages and neutrophils (Fig. 3d, e). We observed a trend for an increased proportion of IFN-γ^+^ CD4^+^ T and reduction of IL-4^+^ CD8^+^ T cells during acute TB for the BCGΔBCG1419c-vaccinated group (Fig. 3f, g), although this did not reach statistical significance. No differences in the proportions of these populations were observed in chronic infection (Supplementary Fig. 2).

During chronic TB, we found that while BCG maintained similar levels of dendritic cells (DCs) as non-vaccinated mice (around 7%, *p* = 0.8669), BCGΔBCG1419c slightly increased the percentage of these cells to around 10% (*p* = 0.0282, Fig. 3b–d). Finally, there was a trend for an increased proportion of IFN-γ^+^ CD4^+^ T cells during acute TB for the BCGΔBCG1419c-vaccinated group (Fig. 3e).

### Vaccination with BCG strains in T2D mice followed by *M. tuberculosis* challenge results in differential production of cytokines from lung cells

**Fig. 4:**
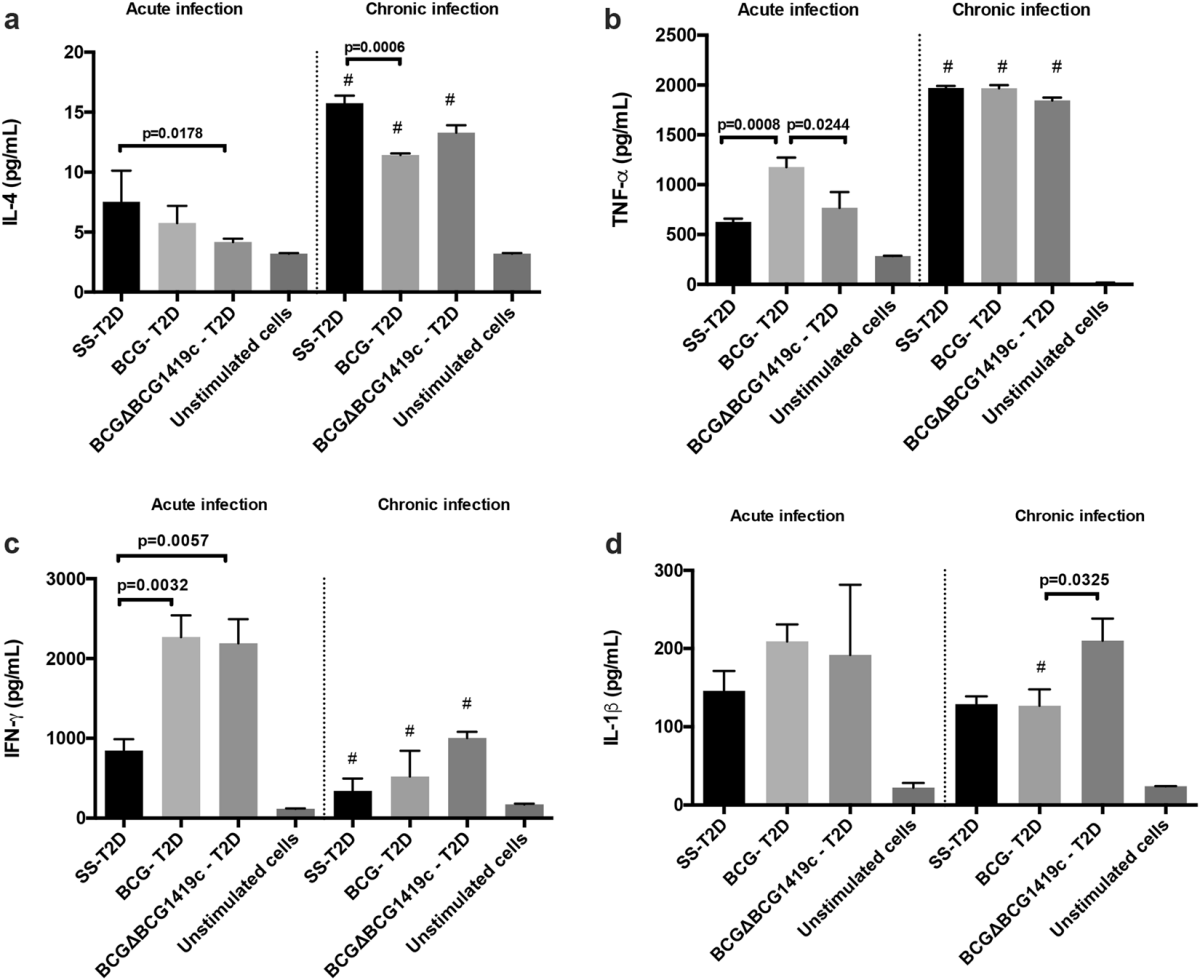
T2D mice vaccinated with BCG strains and infected with *M. tuberculosis* show different production of IFN-γ, TNF-α, IL-1β, and IL-4 during acute or chronic TB. Cells obtained from lungs of all groups were stimulated with PPD during 24 h and the production of cytokines was measured by ELISA (**a**) IL-4, (**b**) TNF-α, (**c**) IFN-γ, (**d**) IL-1β. Data are presented as mean plus standard derivation from four mice per group per time. Group comparisons were *p* < 0.05 were considered different. Data correspond to a representative replicate of two independent in vivo experiments. ***p < 0.0001 in the comparison between groups determined by ANOVA with Bonferroni correction for multiple comparisons; ^#^*p* < 0.05 in the comparison of the same group at 2 and 4 months after infection determined by a two-tailed Student’s *t* test.

After observing changes in immune cells recruitment to lungs, we determined how T2D affect the production of cytokines by lung cells, and found that T2D mice vaccinated with BCG strains responded differently to infection with *M. tuberculosis*. Although during acute TB, both BCG strains induced more IFN-γ (*p* = 0.0032 and *p* = 0.0057 for BCG and BCGΔBCG1419c, respectively), only BCG induced more TNF-α (*p* = 0.0008), and only BCGΔBCG1419c reduced the production of IL-4 (*p* = 0.0178) (Fig. 4).

During chronic TB in T2D mice, TNF-α was the most abundantly produced cytokine regardless of mice being vaccinated or not (Fig. 4b). Both BCG strains induced lower production of IL-4 (*p* = 0.0006) but only vaccination with BCGΔBCG1419c produced a higher induction of IL-1β in lungs compared with BCG-vaccinated mice (Fig. 4d, *p* = 0.0325).

## Discussion

T2D affects several immune mechanisms involved in response to TB, and leads to extended lung pathology in human patients, who also require longer times of antibiotic treatment to result in negative culture from sputa^6,7^. Some reports have shown the response of diabetic animal models to infection with mycobacteria in mice using *M. fortuitum* and BCG^22,23^ as infecting microorganisms, or guinea pigs infected with *M. tuberculosis*^24^. Nevertheless, there is no information reported thus far about how T2D can modify the efficacy of BCG or any other vaccine candidate against TB. Owing to the epidemiological importance of TB-T2D prevalence, the preclinical evaluation of new vaccines against TB conditions, which mimic the TB-T2D comorbidity, may improve our understanding about the efficacy of new vaccine candidates in future clinical studies. Therefore, we contend that efficacy studies of vaccine candidates against TB should consider how does T2D impact protection against this disease. Hence, in this work we endeavored to determine the effect of T2D in the efficacy of protection conferred by BCG and BCGΔBCG1419c against TB, in both acute and chronic stages of this disease.

We were able to reproduce in BALB/c mice several metabolic parameters found in T2D patients, such as increased weight gain, blood glucose, A1C levels, and HOMA insulin-resistance index as compared with control-fed mice (Fig. 1), although we acknowledge that our murine model lacks of fully resembling the physiopathology of human T2D, as we did not find hypertriglyceridemia^25,26^. Nevertheless, we consider this an easily tractable model in which vaccine candidates can be tested, allowing us for the direct comparison of our previously published works conducted in the absence of T2D regarding vaccine efficacy and *M. tuberculosis* pathogenesis, and amenable to evaluate other vaccine candidates.

Interestingly, we found that vaccination with either BCG or BCGΔBCG1419c, or infection with *M. tuberculosis*, decreased the progression of T2D by ameliorating insulin resistance and hyperglycemia. Our findings may correlate, at least to some extent, with reports from other groups showing that BCG reduced insulin resistance in non-T2D mice, coincident with a reduced accumulation of fat^27^, or by increasing their glycolytic flux and downregulation of oxidative phosphorylation, which should reduce glycemia and insulin resistance^28^. In support for a role of mycobacteria-driven increase in glycolytic flux, it was recently reported that BCG vaccine applied to humans with established T1D results in stable and long-term reductions in blood sugar and epigenetic changes in T regulatory cells signature genes for restored tolerance, with both beneficial effects appearing to be driven by a systemic metabolic shift from oxidative phosphorylation towards accelerated and early aerobic glycolysis^29^. Alternatively, it was shown that adipose tissue macrophages are a source of insulin in obese mice^30^, suggesting another possible mechanism that contributes to normalization of blood glucose concentrations in our T2D mice, both vaccinated and/or infected mice. Whether BCG and *M. tuberculosis* have the same effect on glycolysis reported for T1D, now in our T2D model, remains to be determined, as evaluating the role of mycobacteria in T2D was beyond the scope of this work. In addition to differences in glycemic control in T2D mice observed after vaccination with BCG or BCGΔBCG1419c, they produced differences in weight loss at both early and chronic infection, which may result from a complex effect on metabolic changes produced during T2D. The effect of BCG on weight gain or loss in human children is not conclusive^31,32^, therefore the observed effect in T2D mice deserves exploration in future studies using this or similar models to confirm or reject whether this is a T2D-particular phenomenon.

Regarding TB pathology in T2D mice, we observed an increased pneumonia in non-vaccinated mice as compared with both BCG-vaccinated groups (Fig. 2a), with BCGΔBCG1419c significantly reducing it by 50% during chronic TB (Fig. 2b, c), outperforming BCG. Specifically, vaccination with BCG was able to reduce lung immunopathology during acute infection, with a decaying efficacy during chronic infection (as the mean percentage of pneumonia in acute and chronic TB going from ca. 20% to ca. 40%, respectively), while vaccination with BCGΔBCG1419c was effective in reducing lung damage during chronic TB (as the mean percentage of pneumonia in acute and chronic TB going from ca. 35% to ca. 20%, respectively) (Fig. 2b). We also found that both BCG strains were able to reduce *M. tuberculosis* bacillary load in lungs without a significant difference between them (Fig. 2i), despite either BCG or BCGΔBCG1419c inducing different recruitment of immune cells to lungs (Fig. 3) and cytokine production from lung cells (Fig. 4), therefore suggesting that in this particular model, bactericidal mechanisms elicited in response to vaccination with these BCG strains are different, yet in the end, they achieved the same level of control of *M. tuberculosis* burden in lungs. In agreement with these results, it was found that recombinant human lactoferrin reduced lung pathology when used in combination with BCG, and that the sole BCG showed no difference in reducing *M. tuberculosis* load in lungs regardless of lactoferrin supplementation or not^33^. Of note, the authors, experts in TB pathology, recognized that it is the spreading alveolitis, and not the numbers of *M. tuberculosis* bacilli, the event that eventually kills infected models, as it occurs in many humans^33^. In fact, in the early 2000s, it was shown that in vivo lethality following *M. tuberculosis* challenge sometimes dissociated from its replication in organs for strains devoid of some genes, such as *whiB3*^34^, *sigH*^35^, and *sigC*^36^, to mention some examples. We hypothesize that the protection conferred by vaccination with BCGΔBCG1419c against lung pathology in mouse models of TB, already observed in non-diabetic mice^12,15^, might potentially contribute to a more prolonged median survival time of T2D mice with TB as compared with those receiving BCG, which remains a matter of future investigation. Moreover, as sterilization is not achieved in murine models of TB, it could well be that reduction of lung pathology translates into a clinically meaningful phenotype for vaccine efficacy in mice.

Vaccination with BCG or BCGΔBCG1419c strains had a different effect on the average composition of immune cells within lungs of T2D mice. During acute TB, BCG increased the recruitment of T CD4^+^ cells, while BCGΔBCG1419c increased the recruitment of T CD8^+^ and B lymphocytes (Fig. 3c). CD4^+^ T lymphocytes constitutes one of the principal sources of interferon gamma (IFN-γ), a cytokine highly relevant for TB control, and essential for maintaining the structural integrity of the granulomas^37^. In fact, given the important role of IFN-γ-secreting CD4^+^ T cells in animal studies, the main strategy behind the development of many new TB vaccines has been the capacity to promote this type of response^38^. Despite this, how exactly CD4^+^ T cells participate in immunity to TB remains a matter of debate^39,40^. This further suggests that CD4^+^ T lymphocytes are not the single most important component of cell-mediated immunity, which, at least in the context of acute TB and T2D, comes from the fact that BCGΔBCG1419c promoted an increased ratio of T CD8^+^/CD4^+^ lymphocytes (Fig. 3); yet, lung pathology and *M. tuberculosis* load in lungs were close when compared with levels reached after BCG immunization (Fig. 2). Further adding to the role of cells other than T CD4^+^ lymphocytes in control of TB, B cells are known to participate in the formation of granulomas and regulation of the immune response during TB^41,42^. Considering that BCGΔBCG1419c induced the recruitment of B lymphocytes during acute TB in T2D mice, this highlights the need to ascertain whether humoral immunity, per se or in combination with T CD8^+^ cells, plays any role in reduction of TB pathology upon vaccination with this particular vaccine candidate, as opposed to the T CD4^+^-driven response elicited by BCG.

During acute TB in T2D mice, the production of cytokines relevant for TB pathogenesis also responded differently to the BCG strains tested here. On the one hand, BCG led to an increased pro-inflammatory response (TNF-α and IFN-γ) as well as increased anti-inflammatory response (IL-4) (Fig. 4). Conversely, the pro-inflammatory response elicited by BCGΔBCG1419c occurred only via IFN-γ, while a lower anti-inflammatory response was observed (IL-4) (Fig. 4). Considering that TB in diabetic guinea pigs also resulted in an increased pro- and anti-inflammatory response in lungs, mediated by IFN-γ, IL-17A, IL-8, and IL-10 ^24^, this unbalance in pro- and anti-inflammatory response detected in both models of TB-T2D may contribute to both a higher susceptibility to TB and a more severe disease^43^. It has been suggested that effective vaccination against TB should lead to a reduction in the production of IL-4 to confer improved protection against TB^44^, and our results reinforce this notion, particularly in the context of T2D.

During chronic TB, the most significant change in terms of immune cell composition was that BCGΔBCG1419c significantly increased the percentage of DCs compared with BCG-vaccinated and non-vaccinated mice (Fig. 3b–d). DCs infected with *M. tuberculosis* are activated and produce TNF-α, IL-6, IL-1β, and IL-12^45^. DCs infected with BCG maturate and produce IL‐1, IL‐6, IL‐12, IL‐10, and when administered intratracheally in mice, they induced the production of IFN‐γ, leading to a significant protection against aerosol *M. tuberculosis* infection^46^. Moreover, γδ T cells that produce IFN-γ increase production of IL-12 by lung DCs, priming a T CD8^+^ cell response against *M. tuberculosis*^47^. In light of these evidences, it could be hypothesized that DCs might improve the anti-*M. tuberculosis* response induced upon vaccination with BCGΔBCG1419c via pro-inflammatory cytokines, such as IL-1β, and/or other cytokines or chemokines not evaluated in this work.

A reduction of IL-1β, TNF-α, and IL-6 and increased pulmonary inflammation was found when BCG was used to intravenously infect C57BL/6 diabetic mice^23^. Conversely, in our experimental model, lungs from BCGΔBCG1419c-vaccinated mice maintained a higher production of IL-1β in lungs, suggesting that this response could contribute to protection even with low production IFN-γ (Fig. 4).

Compared with parental BCG, the BCGΔBCG1419c vaccine candidate has been shown to have reduced transcription of *groEL1*, *kasA*, *kasB*, *fas*, *fabD*, and *acpM*, genes involved in mycolic acid biosynthesis, as well as reduced transcription of genes encoding for antigenic proteins such as *hspX*^15^. This BCG mutant also produced slighlty less of the antigenic proteins PstS2, HbhA, DnaK, and 35KDa antigen, among other proteins, than wild-type BCG^48^. Taken together, these changes might contribute to explain the differences observed in response elicited to immunization with these BCG strains, yet it would be worth defining which particular components from this vaccine candidates are directly responsible for the differential response in order to better understand immunity triggered by this BCG strain. Also, future studies need to include wider determinations of the response from cells, cytokines, chemokines, and antibodies, to further improve our knowledge of how to achieve control of TB in the context of T2D comorbidity, including changes that occur in the function of immune cells during progression from prediabetic stages to T2D, as well as in reactivation from LTBI likely induced by T2D. Studies of the effect of T2D on macrophage polarization and function of recruited cells to lung will contribute to this aim. Taken together, our results demonstrate that even though T2D involves reduction of some components of a Th1 response and increase of IL-4, vaccination with BCG reduced TB pathology via T CD4^+^ lymphocytes, production of IFN-γ, and TNF-α, being more effective than BCGΔBCG1419c against pneumonia during acute TB. On the other hand, BCGΔBCG1419c was able to confer protection against TB via the maintenance of B and T CD8^+^ lymphocytes during acute TB, favoring the latter as opposed to T CD4^+^ lymphocytes being more attracted to lungs in response to BCG, and by increasing IL-1β production during chronic TB, outperforming BCG in reducing pneumonia in advanced TB-T2D. In summary, more studies of this vaccine candidate are needed to better understand the basis of protection against TB, particularly its chronic phase. Considering that T2D affect antigen presentation by macrophages, activation of monocytes, and chemoattraction of immune cells to lung during infection, BCGΔBCG1419c provides an opportunity to ascertain the role of cells other than macrophages and T CD4^+^ lymphocytes in protection conferred by this vaccine candidate, despite T2D.

## Methods

### Bacterial strains and culture conditions

The BCGΔBCG1419c was previously reported^15^. Mycobacterial strains were cultured in Middlebrook 7H9 broth supplemented with 0.2% glycerol, 10% OADC, and 0.05% Tween 80, at 37 °C, 5% CO_2_, 100 rpm to produce bacteria for vaccination and infection. For lung bacterial counts, serial dilutions were plated onto Middlebrook 7H10 agar supplemented with 0.5% glycerol, 10% OADC, and incubated at 37 °C for 3–4 weeks, colonies were determined by counting colony forming units (CFU).

### Animals

Pathogen-free, male, 3-week-old BALB/c mice were obtained from Instituto Nacional de Ciencias Médicas y Nutrición Salvador Zubirán (*N* = 75). Mice were maintained in a Biosafety Level 3 Animal facility, within vented cages with high-efficiency air-filters, a cycle of light/dark of 12 h, and controlled temperature (21 °C). Food and water were provided ad libitum. The local Animal Ethics Committee of the Instituto Nacional de Ciencias Médicas y Nutrición Salvador Zubirán approved all experiments, which were performed following Mexican guidelines regarding ethical and safe handling of experimental animals: NOM-07-SEMARNAT-SSA1-2002, NOM-033-ZOO-1995, and NOM-062-ZOO-1999.

### Establishment of the T2D mouse model

From its reception, mice were separated into two groups: one fed with an HFD, and the other fed with a balanced, control chow diet (C). HFD was constituted by 45% kilocalories from fat, 34% kilocalories from carbohydrates, and the remaining 21% kilocalories proteins, fiber, vitamins and minerals (Table 1). This diet was prepared weekly in our laboratory and maintained at 4 °C to preserve its components. Control diet was obtained from Pico-Lab laboratories (Rodent Diet 5053).

**Table 1:** Composition of the high-fat diet (HFD) for T2D mice.

Ingredients	Composition per 5 kg (g)	Cat. number
Cistine	9.00	SIGMA 1002645379
Choline	11.50	SIGMA C2004
Vitamins	145.00	MP 960402
Minerals	285.00	MP 960400
Cellulose	86.00	
Soy oil	285.00	Nutrioli™
Dextrin	1500.00	
Saccharose	586.00	Soriana™
Casein	1207.50	
Lard	885.00	

To induce T2D in mice feed with the HFD regime, a single dose of 100 mg/kg of STZ (Sigma, 18883-66-44) diluted in citrate buffer was applied intraperitoneally, at the fourth month after receiving mice (Fig. 1a).

Blood samples were obtained from mice at 0, 2, 4, 8, and 10 months of intervention, and used to determine glucose concentration using a glucometer (Accu check GC 05088810001). The concentration of triglycerides and total cholesterol was determined in sera samples (Unicell DX600, Beckman coulter) and insulin concentration determined by ELISA (Thermo Fisher, EMINS). From total blood, A1C was measured by HPLC (Variant II of BIO-RAD). To measure insulin resistance, the HOMA-IR index was calculated as follows: [insulin concentration (μUI/mL)] × [blood glucose concentration (mmol/L)]/22.6^21^.

### Vaccination and protection against TB in the presence of T2D

Two months after the beginning of HFD feeding, mice that received STZ (T2D) were divided into three groups for vaccination: BCG Pasteur, BCGΔBCG1419c or mock-vaccinated (control) mice (*n* = 25/group) (Fig. 1a). Mice received a single subcutaneous dose of the corresponding BCG strain diluted in sterile saline solution or single sterile saline solution (control). Doses were confirmed to contain (1.4 ± 0.1) × 10^4^ CFU by plating onto 7H10 plates.

Pulmonary bacillary burdens and tissue damage (pneumonia) were determined at 2 and 4 months post challenge with *M. tuberculosis* H37Rv in vaccinated and control, non-vaccinated mice. The right lung was frozen up to be used for the determination of bacillary burdens by CFU counting. After defrosting, lungs were mechanically disaggregated in 1 mL of 0.05% Tween 80 (Sigma 9005-65-6) in phosphate-buffered saline (PBS) solution. Serial dilutions were plated in duplicate onto 7H10 agar plates and incubated for 14–21 days for colony counting. Overall, 6–7 lungs per group/time post infection were used for CFU enumeration.

To evaluate lung damage, the left lung was perfused with absolute ethanol after euthanasia and fixed in the same solution for at least 48 h. Lungs were included in paraffin blocks. Then, 4 μm lung sections were obtained, stained with hematoxylin and eosin and evaluated. The determination of the percentage of pneumonic area was performed using the automated histology software Leica Application Suite X. Three lungs per group of different mice were evaluated and determination was made in duplicate.

### Evaluation of cellular immune response after infection in the presence of T2D

The left lung of each mouse was used to separate cells by using type II Collagenase (Gibco 9001-20-1) and mechanical disaggregation, to then culture them in 24-well plates with PPD 1 μg/mL and antibiotic solution at 37 °C, 5% CO_2_ for 24 h. Supernatant was used to determine by ELISA TNF-α (BD OprEIA, 558534), IL-1β (BD OprEIA, 559603), IL-4 (BD OprEIA, 555232) and IFN-γ (BD OprEIA, 555138) according to the manufacturer’s protocols. Four supernatants per group were processed in duplicate to determine the concentration of cytokines secreted to the medium. In addition, after stimulation with PPD, 1 × 10^6^ cells per lung were separated and used for extracellular staining of surface markers including a viability staining, as indicated in supplementary methods. After permeabilization and fixation of cells (Invitrogen, 00-5523-00), we performed intracellular staining with anti-IFN-γ-BV786, anti-TNF-α-AF488, anti-IL-12-PE, and anti-IL-4-BV421. Finally, labeled cells were washed with PERM and resuspended in 500 μL of 4% paraformaldehyde. Cells were maintained at 4 °C in dark up to data obtaining before acquisition by flow cytometry. Flow cytometry data acquisition was performed in a BD Fortessa cytometer using FACS-DIVA software in the next 24 h after labeling. Data obtained from 100,000 total cells per lung were analyzed with FlowJo v.10 software to determine the percentages of B cells (CD19^+^ cells), macrophages (CD14^+^ CD11b^+^ F4/80^+^ cells), CD11b^+^ DCs (CD14^+^ CD11b^+^ F4/80^-^ cells), neutrophils (CD14^low^ CD11b^+^, Ly6G^+^), CD3^+^ CD4^+^, and CD3^+^ CD8^+^ T cells that produced IFN-γ, TNF-α, IL-12, and IL-4 in response to stimulation with PPD. Identification of cell populations is shown in Fig. 3a. Four lungs per group/time post infection were processed. Detailed protocols for lung cell separation and flow cytometry are provided in Supporting information as supplementary methods for FACS (Supplementary Fig. 3).

### Postinfection histological analysis

A qualified pathologist analyzed images of the hematoxylin and eosin-stained sections from lungs without prior knowledge of the groupings. A scoring system (0 = no apparent changes to 5 = severe changes) that involved the examination of lungs for peribronchiolitis, perivasculitis, alveolitis, “Granuloma” formation, and degree of necrosis was used to give a total lung score for lungs from each mouse. Lesions were assessed as reported before^15,49^; Briefly, the number of lesions apparent in a section was counted and the percentage of involved parenchyma estimated. The following features were assessed individually: peribronchiolitis, perivascular leukocyte infiltration, perivasculitis, alveolitis, “granuloma” formation (i.e., granulomatous inflammation), and necrosis on a scale of 0–5 [0 = within normal limits (no change); 1 = minimal changes; 2 = mild changes; 3 = moderate changes; 4 = marked changes; 5 = very severe changes].

### Statistical analysis

Data are presented as means with standard deviations, median and ranges, or median with standard deviation. Comparison among groups were performed in Prism v.7 using a one way-ANOVA analysis with a Tukey’s analysis pos hoc analysis for multiple comparisons between groups at the same time, and with a double-tailed Student’s *t* test to compare data from the same group at different times. Group comparisons where *p* < 0.05 were considered different. Categorized data (histological analyses) were analyzed with a H Krustal–Wallis test using SPSS version 25 (*α* = 0.05). In all cases, *p* value were adjusted for multiple comparisons with Bonferroni correction. Data presented here correspond to a representative replicate out of two independently performed experiments.

### Reporting summary

Further information on research design is available in the Nature Research Reporting Summary linked to this article.

## Supplementary information

Supplementary Information

Reporting summary

## Data Availability

The authors declare that data supporting the findings of this study are available within the article and its Supplementary Information files.
