# Author Correction: BCG and BCGΔBCG1419c protect type 2 diabetic mice against tuberculosis via different participation of T and B lymphocytes, dendritic cells and pro-inflammatory cytokines

**DOI:** 10.1038/s41541-020-00250-y

**Published:** 2020-10-20

**Authors:** Cristian Alfredo Segura-Cerda, Brenda Marquina-Castillo, Vasti Lozano-Ordaz, Dulce Mata-Espinosa, Jorge Alberto Barrios-Payán, Manuel O. López-Torres, Michel de Jesús Aceves-Sánchez, Helle Bielefeldt-Ohmann, Rogelio Hernández-Pando, Mario Alberto Flores-Valdez

**Affiliations:** 1 Doctorado en Farmacología, Universidad de Guadalajara, Sierra Mojada 950, Col. Independencia Oriente, 44340 Guadalajara, Jalisco Mexico; 2 Biotecnología Médica y Farmacéutica, Centro de Investigación y Asistencia en Tecnología y Diseño del Estado de Jalisco, A. C., Av. Normalistas 800, Col. Colinas de la Normal, 44270 Guadalajara, Jalisco México; 3 Laboratorio de Patología Experimental. Instituto Nacional de Ciencias Médicas y Nutrición Salvador Zubirán, Vasco de Quiroga 15, Belisario Domínguez sección 16, Tlalpan, Ciudad de México, México; 4 School of Chemistry & Molecular Biosciences, University of Queensland St. Lucia Campus, St Lucia, QLD 4072 Australia

**Keywords:** Vaccines, Live attenuated vaccines

Correction to: *npj Vaccines* 10.1038/s41541-020-0169-6, published online 12 March 2020

A panel was missing in Fig. 3 in the original version of this Article. The figure and legend have now been corrected in the PDF and HMTL versions of the Article.

