# Supplementary material for: BCG and BCGΔBCG1419c protect type 2 diabetic mice against tuberculosis via different participation of T and B lymphocytes, dendritic cells and pro-inflammatory cytokines
Source: NPJ Vaccines. 2020 Mar 12;5:21. doi: 10.1038/s41541-020-0169-6 (PMC7067831; doi:10.1038/s41541-020-0169-6)
Supplement: Supplementary file 1 — Supplementary Information [file 41541_2020_169_MOESM1_ESM.pdf]

Supplementary Table 1. Food intake of HFD feed mice before infection in vaccinated groups.

Group	Food intake (g/mice-week)	*p* SS	*p* BCG Pasteur
SS	2.21 ± 0.36	-	
BCG Pasteur	2.34 ± 0.73	0.7500	-
BCGΔBCG1419c	2.33 ± 0.14	0.5572	0.9794

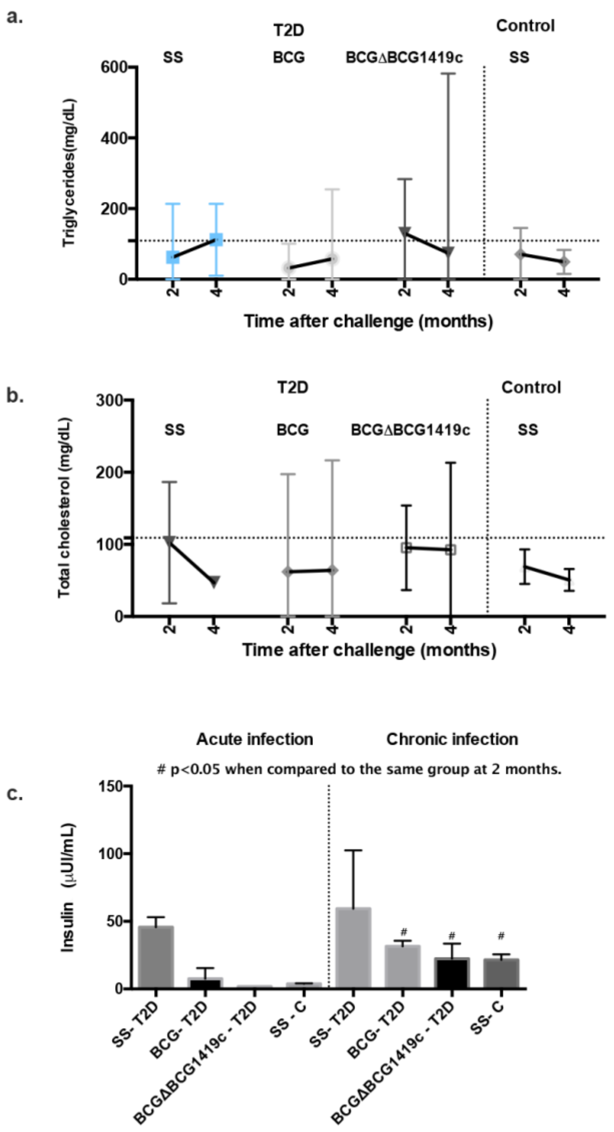

Supplementary Figure 1. Metabolic markers of progression to T2D. (a) Triglycerides, **(b)** Total cholesterol, and **(c)** Insulin. Data are presented as median and range of 5 mice per time evaluated (A and B), or media and standard derivation (C). Group comparisons were $p<0.05$ were considered different. Data correspond to a representative replicate of two independent *in vivo* experiments. *** $p<0.0001$ in the comparison between groups determined by ANOVA with Bonferroni correction for multiple comparisons; # $p<0.05$ in the comparison of the same group at two and four months after infection determined by a two-tailed Student´s T test.

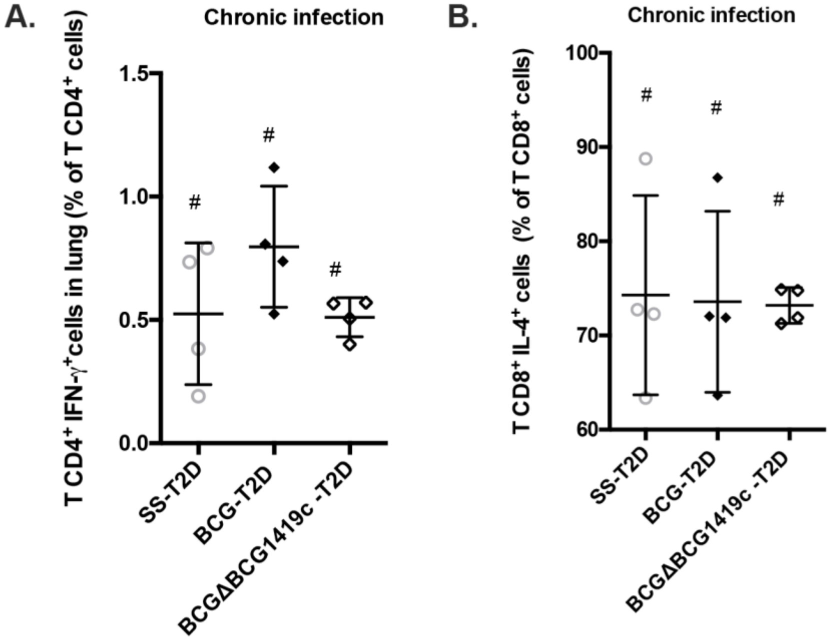

Supplementary Figure 2. T cell immune response in lungs. The percentage of T **(a)** $CD4^{+}$ or **(b)** $CD8^{+}$ lymphocytes producing IFNγ or IL-4, respectively, are shown. Dots represent individual values and the central line the mean. Data correspond to a representative replicate of two independent *in vivo* experiments. *** $p<0.0001$ in the comparison between groups

determined by ANOVA with Bonferroni correction for multiple comparisons; # $p<0.05$ in the comparison of the same group at two and four months after infection determined by a two-tailed Student´s T test.

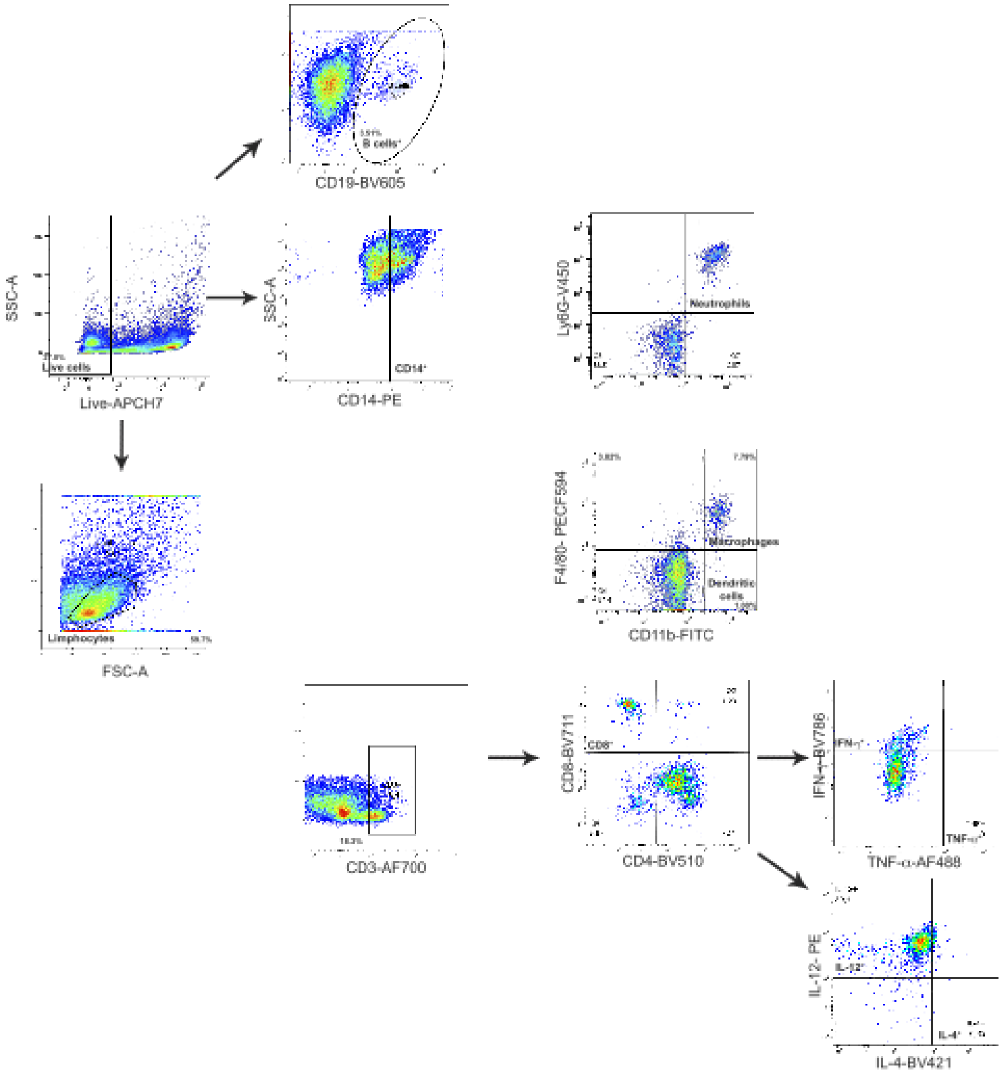

Supplementary Figure 3. Gating strategy applied to identify each cell population. Cells were first gated by staining with APCH7-Fixable viability stain (lower than $1x10^3$). Live cells were then divided into four groups of analysis: Group 1. From live cells, CD19$^+$ cells were

chosen (limit at $1x10^3$) to recognize B cells ($CD19^+$); Group 2. From live cells, $CD14b^+$ cells were chosen (limit at $1x10^4$). Then cells were divided in a double-selection graph including Ly6G-V50 (limit at $1.5x10^2$) and CD11b (limit at $1x10^2$), to recognize neutrophils ($Ly6G^+CD11b^+$); Group 3. From live cells, $CD14b^+$ cells were chosen (limit at $1x10^4$). Then cells were divided in a double-selection graph including F4/80-PECF594 (limit at $9x10^2$) and CD11b (limit at $5X10^2$), to recognize macrophages ($F480^+CD11b^-$) and dendritic cells ($F480^-CD11b^+$); Group 4. From live cells, lymphocytes were gated in the typical region of these cells, in a FSSC-A/FSC-A graph. Then, from lymphocytes, $CD3^+$ cells were chosen by gating $AF700^+$ cells (limit at $1x10^3$). Afterwards cells were gated into a double selection graph including CD8-BV711 (limit at $4x10^3$) and CD4-BV510 (limit at $5x10^3$). $CD4^+$ and $CD8^+$ T cells were chosen in this graph to be analyzed separately: 4a. $CD3^+CD4^+$ T cells were divided in a double selection graph including IFN-BV786 (limit at $1x10^4$) and TNF-AF488 (limit at $1x10^4$) to be $CD3^+CD4^+IFN\gamma^+$ or $CD3^+CD4^+TNF\alpha^+$, 4b. $CD3^+CD4^+$ T cells were divided in a double selection graph including IL-12-PE (limit at $1x10^3$) and IL-4-BV421 (limit at $1x10^4$) to be $CD3^+CD4^+IL\text{-}12^+$ or $CD3^+CD4^+IL\text{-}4^+$.

Supplementary method for FACS.

After stimulation with PPD, $1x10^6$ cells per lung were separated and placed into cytometry tubes to wash them with PBS. Then, cells were incubated with 0.5 µg of FC purified anti-mouse CD16/CD32 (TONBO, 70-0161) in 100 µL of PBS for 10 min at 4 ºC. After this, cells were washed with 1 mL of PBS and viability staining was performed by incubating cells during 15 min with 80 µL of 1:79 Flexible Viability staining 750 (BD 565358) diluted in PBS at 4 ºC. Next, for extracellular staining of surface markers, cells were incubated with anti-CD11b-FITC, anti-CD14-PE, anti-F4/80-PECF594, anti-Ly6G-V450, anti-CD3-AF700, anti-CD4-BV510, anti-CD8-BV71 each diluted at

1:99 in 20 μL of PBS. To stop this step, cells were washed with 2% bovine serum albumin (Calbiochem, 12660) in PBS and then a permeabilization and fixation step was performed with a buffer set according to manufacturer´s instructions (Invitrogen, 00-5523-00). For intracellular staining of cytokines, fixed and permeabilized cells were incubated for 30 min at 4 ºC with anti-IFN-γ-BV786, anti-TNF-α-AF488, anti-IL-12-PE and anti-IL-4-BV421 each at 1:99 dilution in PERM buffer from the above-mentioned buffer set. Finally, labeled cells were washed with PERM and resuspended in 500 μL of freshly prepared 4% paraformaldehyde. Cells were maintained at 4 ºC in dark up to data obtaining before acquisition by flow cytometry.

Flow cytometry data acquisition was performed in a BD Fortessa cytometer using FACS-DIVA software in the next 24 hours after labeling. Data obtained from 100,000 total cells per lung were analyzed with FlowJo v.10 software to determine the percentages of B cells ($CD19^+$ cells), macrophages ($CD11b^+$ $CD14^+$ $F4/80^-$ cells), $CD11b^+$ dendritic cells ($CD11b^+$ $CD14^+$ $F4/80^+$ cells), neutrophils ($CD11b^+$, $CD14^{low}$ $Ly6G^+$), $CD3^+$ $CD4^+$ and $CD3^+$ $CD8^+$ T cells that produced IFN-γ, TNF-α, IL-12, and IL-4 in response to stimulation with PPD. Identification of cell populations is shown in Figure 3A**.** Four lungs per group/time post-infection were processed.
